# Sustained efficacy of collars containing 10% w/w imidacloprid and 4.5% w/w flumethrin (Seresto^®^) in dogs against laboratory challenge with *Haemaphysalis longicornis* (Neumann, 1901) ticks

**DOI:** 10.1186/s13071-022-05206-w

**Published:** 2022-03-05

**Authors:** Leon Meyer, Nouha Lekouch, Gertraut Altreuther, Bettina Schunack, Matthias Pollmeier

**Affiliations:** 1 Clinvet Morocco, Mohammedia, Morocco; 2 Elanco Animal Health, Bayer Animal Health GmbH, Monheim, Germany

**Keywords:** *Haemaphysalis longicornis*, Dogs, Imidacloprid, Flumethrin, Sustained efficacy

## Abstract

**Background:**

*Haemaphysalis longicornis* ticks are reported on dogs from an increasing geographic range. This study aimed to determine the sustained efficacy of Seresto^®^ collars (imidacloprid/flumethrin) against experimental infestations of *H. longicornis* in dogs.

**Methods:**

Twenty-four Beagle dogs previously assessed for their suitability to harbor ticks were included in the study and randomized into three groups of eight dogs each. Two of the groups were treated with collars at different time points: at the first tick infestation, dogs in group 1 had already worn collars for 92 days, while dogs in group 2 had received collars only on the previous day, thus allowing evaluation of two different treatment durations at the same point in time. Infestation of the treated groups was conducted at 1, 7, 28, and 56 days (group 2) and 92, 119, 147, 168, 196, 227, and 238 days (group 1) after collar placement. Group 3 served as untreated control and was infested whenever the dogs of the other two groups were infested. Infestations were conducted using 50 viable, adult, unfed female ticks of a US isolate of *H. longicornis* per dog. Ticks were removed and counted 48 h after each infestation. Health and body weight of the dogs were monitored throughout the study. The efficacy against ticks was calculated for groups 1 and 2 based on arithmetic mean values at each assessment day according to Abbott’s formula. The mean post-treatment *H. longicornis* tick counts were compared statistically between treatments, using an analysis of variance with a treatment effect untransformed tick count.

**Results:**

Dogs in the control group were adequately infested at all tick counts. Efficacy was 88.2% on day 3, however well above 90% (i.e., 98.3 to 100%) at all other time points up to day 240. Statistical analysis confirmed significantly different live tick counts (*P* < 0.001) between the treated groups and the control group at all time points.

**Conclusions:**

The 8-month sustained acaricidal efficacy demonstrated by the Seresto^®^ collar (imidacloprid/flumethrin) provides a reliable strategy against *H. longicornis* infestations in dogs.

**Graphical Abstract:**

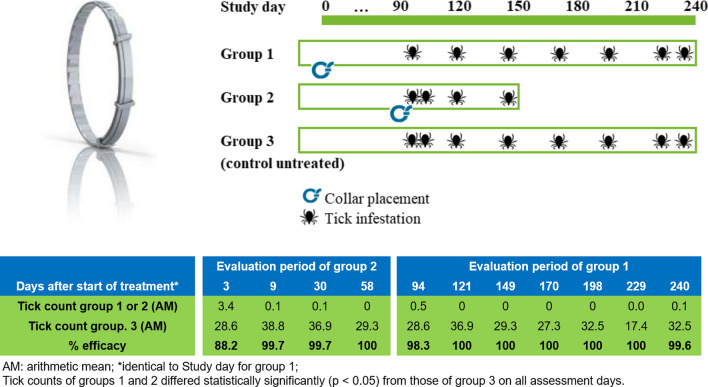

## Background

Tick infestations pose serious health problems to dogs. The longhorned tick, *Haemaphysalis longicornis* (Neumann, 1901), is a three-host ixodid tick (Ixodida: Ixodidae) native to eastern Asia that was subsequently reported from Australia, New Zealand, and numerous Pacific Islands countries [1]. Rainey et al. [2] first reported this species infesting a sheep from New Jersey (USA) in 2017, subsequently increasingly reported from other states in the USA [3–5]. Species determination of historical samples confirmed the presence of this species years earlier, with ticks collected from a deer in West Virginia in 2010 and a dog in New Jersey in 2013 retrospectively identified as *H. longicornis* [6]. The presence of *H. longicornis* in the USA is however considered an emerging disease threat representing a potentially devastating ectoparasite of domestic animals and a vector of both human and veterinary pathogens [6]. Phylogenetic analysis of the sequence of the complete mitochondrial genome (mt-genome) from two *H. longicornis* ticks recently collected in the state of New York (USA, in 2020) showed that the mt-genome clustered with those of other *H. longicornis* identified in China [7]. A meta-analysis of pooled data from a Chinese field survey and global published data concluded that this tick species, known to feed on a variety of domestic and wild animals and humans, was associated with a variety of pathogens, including seven species of spotted fever group rickettsiae, seven species in the family of Anaplasmataceae, four genospecies in the complex *Borrelia burgdorferi* sensu lato, two *Babesia* species, six species of virus (including bocavirus spp. and tick-borne encephalitis virus), and *Francisella*, *Bartonella*, *Coxiella*, and *Toxoplasma*, though mainly reported in eastern Asia [8]. It has also been reported to cause red meat allergy [9]. Predictive ecological niche modeling revealed that *H. longicornis* might affect more extensive North American regions, but suitable habitats might also include Europe, South America, and Africa, where the tick has not yet been recorded [8, 10]. The observed rapid geographical spread of *H. longicornis* is believed to be partly due to reproduction via parthenogenesis [6], their ability to tolerate a wide range of environmental temperatures [11], and tick larvae infesting migratory birds [10]. A study on seasonal distribution from New Jersey suggests that *H. longicornis* is capable of establishing larger populations than other tick species. Peak *H. longicornis* densities for larvae, nymphs, and adults were reported to be 286, 20, and 28 times as high as than those of *I. scapularis*, respectively [12]. Wildlife and tick sampling in Virginia identified 18 different individual hosts and approximately 60% of environmental ticks as being *H. longicornis* [13]. Animal transport, such as infested dogs traveling with their owners, is also of concern. Once established populations exist, they are there to stay, and strategies must focus on mitigating spread, monitoring for emerging diseases, developing multisectoral responses [5], and increase awareness [14, 15].

In this context, the use of effective acaricides on dogs is important to first protect these animals against the effects of ectoparasitic infestations, but secondly to limit the dispersion of this invasive tick species. Collars containing 10% w/w imidacloprid and 4.5% w/w flumethrin, (Seresto^®^) have previously been shown to be efficacious against a variety of tick species including *Ixodes*, *Rhipicephalus*, *Amblyomma*, and *Dermacentor* spp. The present study was performed to assess the sustained efficacy of such collars against the tick *H. longicornis*.

## Methods

### Animals and husbandry

Purpose-bred Beagle dogs approximately 29 to 44 months of age and weighing 9.5 to 13.2 kg were included in the study. Twenty-four dogs, 11 females and 13 males, that were vaccinated against canine distemper, infectious hepatitis, parvovirosis, infectious laryngotracheitis, parainfluenza, leptospirosis, and rabies were dewormed prior to initiation of the study and did not harbor resident tick or flea infestations at the initiation of the study. Dogs had not been treated with a long-acting topical or systemic acaricide/insecticide whose claimed duration of efficacy had not expired for at least 2 months. Dogs were identified by electronic transponders with unique alphanumeric codes.

Dogs were generally housed in pairs within groups, except for days between tick infestation and tick counts, where the dogs were individually housed in indoor enclosures that conformed to accepted animal welfare guidelines and ensured no direct contact between dogs of different groups. During periods when not infested with parasites, dogs of the same sex and from the same group were allowed to access and interact in a social exercise area. Dogs were acclimated to these conditions for at least 7 days prior to treatment. Commercial, dry, canine feed was offered once daily for the duration of the study according to manufacturer’s recommendation. Water was available ad libitum from stainless steel bowls that were replenished at least twice daily. All dogs were given a physical examination to ensure that they were in good health at enrollment and suitable for inclusion in the study. General health observations of each dog were performed daily throughout the study.

### Design

This study was a negative controlled, randomized, non-blinded efficacy study against ticks and was conducted in accordance with the EMEA antiparasitic guideline for dogs and cats [16] as well as Good Clinical Practice [17].

**Fig. 1:**
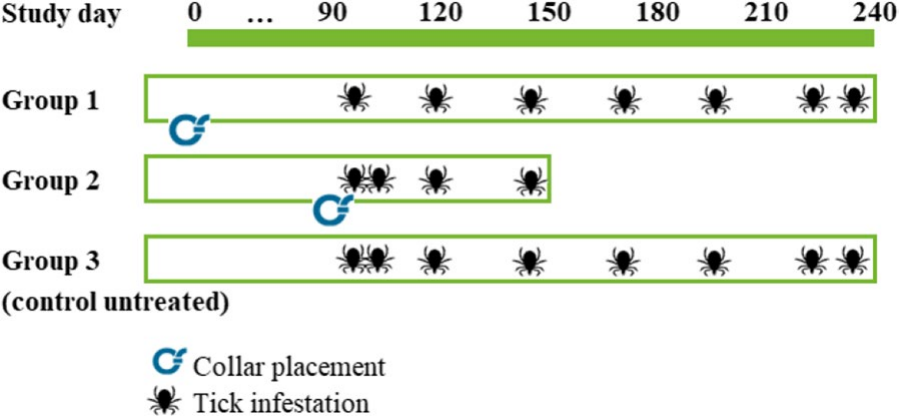
Schematic overview of the study design

Dogs previously assessed for their suitability to harbor ticks were included in the study and randomized based on body weight within sex into three groups of eight dogs each. Two of the groups were treated with collars at different time points: at the first tick infestation, dogs in group 1 had already worn collars for 92 days, while dogs in group 2 had received collars only on the previous day, thus allowing evaluation of two different treatment durations at the same point in time. Infestation of the treated groups was conducted at 1, 7, 28, and 56 days (group 2) and 92, 119, 147, 168, 196, 227, and 238 days (group 1) after collar placement (Fig. 1). Group 3 served as untreated control and was infested whenever the dogs of the other two groups were infested. Tick counts were conducted 48 h after infestations. Dogs in group 1 covered efficacy from 3 to 8 months after collar placement (persistent efficacy), whilst dogs in group 2 covered efficacy from collar placement up to the time where efficacy evaluation of group 1 started (persistent efficacy).

### Treatment

The dogs either received a collar (groups 1 and 2, imidacloprid 10% w/w/flumethrin 4.5% w/w, Seresto^®^) or remained untreated (group 3). Collars were placed and fastened around the dog’s neck according to label instructions, so that it was possible to insert only two fingers next to each other between the collar and the neck when fastened. Excess collar was pulled through the collar’s loops, and any excess length extending beyond 2 cm was clipped.

During the study, the dogs were observed daily during facility cleaning activities for correct collar fit. In case a collar was accidentally lost by a dog, it was immediately reapplied. If a dog’s collar was completely lost or destroyed, the dog would have been removed from the study.

### Tick infestations and assessments

Each dog was artificially infested with 50 viable female *H. longicornis* on the days as set out in Fig. 1. Ticks were adult, unfed, and had molted at least 1 week prior to being used. A laboratory-bred parthenogenetic strain originally sourced from Virginia (USA) in October 2018 was used for the artificial infestations.

To facilitate tick infestation, dogs were sedated with xylazine (Xylased^®^, Bioveta) administered intramuscularly at a dose rate of 2 mg/kg body weight and placed in a dark infestation chamber (50 cm × 50 cm × 90 cm) for at least 4 h following infestation. Ticks were placed on the lower chest of dogs in lateral recumbency and not further manipulated. To facilitate darkness, each infestation chamber was covered with a drape, made from breathable material.

Counting and removal of ticks were performed at 48 (± 2 h) after infestation. Ticks were found by direct observation following parting of the hair coat and palpation of the entire body. Following this, each dog was combed to ensure that all ticks were counted and removed. Ticks were counted and categorized as dead or live, attached or free.

### Statistical analysis

The individual dog was the experimental unit. The efficacy against ticks was calculated based on percent reduction in the arithmetic mean (AM) live (female and male) tick counts relative to control using Abbott’s formula:

$$ {\text{Efficacy}}\left( \% \right){\text{against}}\;{\text{ticks}} = 100 \times \left( {{\text{Mean}}\;{\text{count}}\;{\text{control}}{-}{\text{ Mean}}\;{\text{count}}\;{\text{treated}}} \right)/{\text{Mean}}\;{\text{count}}\;{\text{control}}{.} $$

To statistically support the validity of the efficacy results, the mean post-treatment *H. longicornis* tick counts were compared between treatments, using an analysis of variance (ANOVA) with a treatment effect untransformed tick count. Comparison was pairwise to the control group for each time point, at a two-sided 5% level of significance.

Body weight (BW) and hair length obtained during acclimation were compared between the groups using a one-way ANOVA (Proc GLM procedure in SAS version 9.4) with a treatment effect and assuming a normal distribution of the data, to evaluate their homogeneity at the time of inclusion.

Efficacy was claimed if the following criteria were met: (1) at least six adequately infected dogs in the control group (> 25% retention for ticks [≥ 13 live ticks] [16]), (2) adulticidal efficacy ≥ 90%, and (3) significant difference between the treated and control groups. SAS version 9.4 software was used for all analyses.

## Results

The groups were demonstrated to be homogeneous with regard to dog BW and hair length (*P* > 0.6) at the initiation of the study. The control dogs were adequately infested on all assessment days, with AM tick counts of 17.4 to 38.8. Early efficacy (day 1, group 2 assessment) was 88.2%. Persistent efficacy determined from both treated groups (1 and 2) ranged from 98.3 to 100% on all the assessment days (Table 1). The numbers of ticks collected from the dogs at each count were significantly higher in the control group 3 than in the treated groups 1 and 2 at all time points (*P* < 0.001).

**Table 1:** Arithmetic mean live *Haemaphysalis longicornis* counts and ranges for control dogs and dogs treated with Seresto^®^ and percent efficacy relative to control at 48 h after infestations

Day	Control				Seresto^®^				Efficacy compared to control	
	Group	Mean	Range	Dogs with ticks	Group	Mean	Range	Dogs with ticks	%Efficacy^a^	Test statistic
3	3	28.6	14–43	8/8	2	3.4	0–7	5/8	88.2	*t*_(14)_ = 6.47, *P* < 0.0001
9	3	38.8	30–50	8/8	2	0.1	0–1	1/8	99.7	*t*_(14)_ = 18.41, *P* < 0.0001
30	3	36.9	27–45	8/8	2	0.1	0–1	1/8	99.7	*t*_(14)_ = 20.18, *P* < 0.0001
58	3	29.3	17–37	8/8	2	0	0–0	0/8	100	*t*_(14)_ = 11.74, *P* < 0.0001
94	3	28.6	14–43	8/8	1	0.5	0–2	3/8	98.3	*t*_(14)_ = 7.56, *P* < 0.0001
121	3	36.9	27–45	8/8	1	0	0–0	0/8	100	*t*_(14)_ = 20.29, *P* < 0.0001
149	3	29.3	17–37	8/8	1	0	0–0	0/8	100	*t*_(14)_ = 11.74, *P* < 0.0001
170	3	27.3	19–35	8/8	1	0	0–0	0/8	100	*t*_(14)_ = 13.46, *P* < 0.0001
198	3	32.5	25–40	8/8	1	0	0–0	0/8	100	*t*_(14)_ = 16.95, *P* < 0.0001
229	3	17.4	7–40	8/8^b^	1	0	0–0	0/8	100	*t*_(14)_ = 4.59, *P* = 0.0004
240	3	32.5	15–45	8/8	1	0.1	0–1	1/8	99.6	*t*_(14)_ = 9.97, *P* < 0.0001

^a^Pairwise comparison of tick counts between control and treated groups statistically significantly different at all time points (*P* < 0.001)

^b^On day 229, two out of eight dogs presented with < 13 ticks (7 and 8 ticks, respectively)

There were no adverse reactions related to treatment with Seresto^®^. During general health observations on day 4, one dog in group 1 presented with loose feces. The condition had resolved on day 5 without medical intervention. One dog in the control group exhibited depression and had a low body condition score (BCS 2.5) on day 225. The diet of this animal was adjusted and additional socialization introduced, which led to resolution of the condition.

## Discussion

Seresto^®^ demonstrated excellent 48-h efficacy against *H. longicornis* in dogs, preventing new infestations from occurring for at least 8 months after collar placement. The day 3 results in treatment group 2 did not meet the current efficacy requirements [16] but were significantly different from controls. Furthermore, four of eight dogs in group 2 presented with tick counts ranging from 0 to 1 on day 3. The release kinetics of the collar matrix and resulting body surface distribution depend on several factors that also include dog skin characteristics [18]. This may explain the individual differences observed during the early phase of release and onset of efficacy when ticks may be located distant from the neck.

The tick strain used was demonstrated vigorous as shown by the high average retention rates in the untreated control, group 3, ranging from 35 to 77%. There were at least six dogs with 13 or more live ticks on all occasions exceeding the EMEA [16] minimum adequate infestation requirement of 25% and validating the experimental model. All of the eight control dogs had retention rates that ranged from 28 to 100% on all occasions, except for day 229. On the latter day, two out of eight dogs presented with < 13 ticks (7 and 8 ticks, respectively) at the 48-h count for unknown reasons. These dogs retained 50 and 70% of ticks, respectively, at the following infestation (day 240), and their tick retention rate throughout the earlier phases of the study was inconspicuous (Table 2).

**Table 2:** Adequacy of tick infestation in the control group

Animal	Group	Retention rate (%) on study day …							
		94	100	121	149	170	198	229	240
1	3	56	72	90	72	70	68	80	62
2		58	74	54	62	38	70	42	30
3		60	76	76	74	46	66	28	74
4		80	88	68	34	50	50	16	70
5		62	72	72	54	46	52	14	50
6		28	76	80	48	68	58	46	78
7		86	100	76	72	62	76	26	66
8		28	60	74	52	56	80	26	90
	AM	57	77	74	59	55	65	35	65
	Min	28	60	54	34	38	50	14	30
	Max	86	100	90	74	70	80	80	90

*AM* arithmetic mean, *Min* minimum, *Max* maximum

*Haemaphysalis longicornis* represents a new and emerging disease threat, and further research in the characterization of the tick’s biology and ecology are warranted. The wide host range including rodents [13], ungulates, lagomorphs, carnivores, birds, and humans increases the likelihood that this tick will broaden its geographical reach via dispersion on animal hosts [1, 2, 11]. Determination of vector competency is of high importance and should include testing for potential indigenous and exotic pathogens. This highlights the potential risks and consequences of invasive tick species of medical or veterinary importance, which, once established, are difficult to control. The use of effective acaricides on dogs that are minimally impacted by owner compliance—i.e., a collar applied in spring will provide season-long efficacy—is important to first protect these animals against the effects of ectoparasitic infestations but secondly to limit the dispersion of this invasive tick species.

## Conclusions

The 8-month sustained acaricidal efficacy demonstrated by the combination of imidacloprid and flumethrin in Seresto^®^ provides a reliable strategy against *H. longicornis* infestations in dogs.

## Data Availability

The dataset supporting the conclusions of this article is included within the article.

## Abbreviations

AM: Arithmetic mean
BCS: Body condition score
BW: Body weight
GmbH: Gesellschaft mit beschränkter Haftung (limited liability company)
h: Hours
kg: Kilogram
max: Maximum
mg: Milligram
min: Minimum
